# Temperature and Cognition in ELSA

**DOI:** 10.1093/geroni/igaf122.045

**Published:** 2025-12-31

**Authors:** Paola Zaninotto, Shaun Scholes, Jessica Gong

**Affiliations:** University College London, London, England, United Kingdom; University College London, London, England, United Kingdom; University College London, London, England, United Kingdom

## Abstract

Understanding the relationship between temperature and cognitive function is crucial, especially in older populations, as extreme temperatures may exacerbate cognitive decline and affect overall health. This study explores the longitudinal association between temperatures and cognitive function in a large, nationally representative sample of individuals aged 50 and over, participants in the English Longitudinal Study of Ageing (ELSA). Using nine waves of data collected between 2002 and 2018, temperatures were linked to participants’ residential addresses to assess the impact of temperature variations on cognitive performance. We also examined whether the effect of temperature on cognitive function was more pronounced in vulnerable individuals, including those with pre-existing health conditions. Our findings suggests that repeated exposure to extreme temperatures has an effect on cognitive decline, highlighting the need for tailored public health interventions for older populations.

